# Supplementary material for: Unraveling factors responsible for pathogenic differences in Lassa virus strains
Source: bioRxiv. 2024 May 21:2024.05.21.595091. Preprint. [Version 1] doi: 10.1101/2024.05.21.595091 (PMC11142057; doi:10.1101/2024.05.21.595091)
Supplement: Supplement 1 [file NIHPP2024.05.21.595091v1-supplement-1.pdf]

bioRxiv preprint doi: https://doi.org/10.1101/2024.05.21.595091; this version posted May 21, 2024. The copyright holder for this preprint (which was not certified by peer review) is the author/funder, who has granted bioRxiv a license to display the preprint in perpetuity. It is made available under aCC-BY 4.0 International license.

Supporting information

Table S1. Complete blood count in guinea pigs infected with LASVs at 11 d.p.i.

Table S2. Blood clinical chemistry in guinea pigs infected with LASVs at 11 d.p.i.

Table S3. Immunological transcription analysis in PBMCs

Fig. S1 Body weight and temperature change in guinea pigs infected with 10^4 PFU of LASV LF2384 or LF2350.

bioRxiv preprint doi: https://doi.org/10.1101/2024.05.21.595091; this version posted May 21, 2024. The copyright holder for this preprint (which was not certified by peer review) is the author/funder, who has granted bioRxiv a license to display the preprint in perpetuity. It is made available under aCC-BY 4.0 International license.

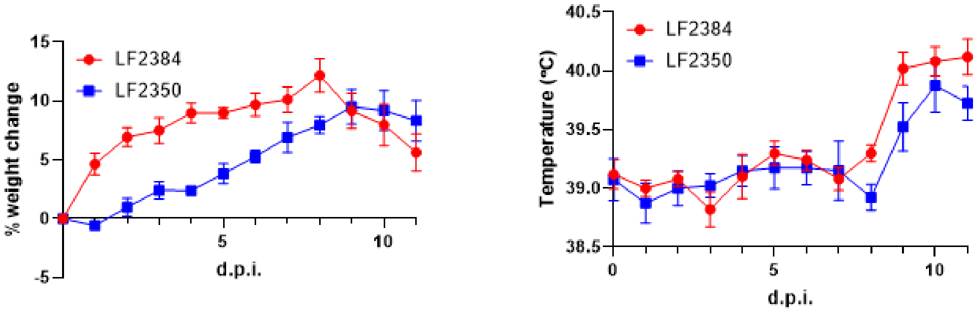

Fig. S1 Body weight and temperature change in guinea pigs infected with 10^4 PFU of LASV LF2384 or LF2350.

Hartley guinea pigs were inoculated with 10^4 PFU of LASV LF2384 or LF2350 intraperitoneally. Body weight (left) and temperature (right) were measured daily until 11 d.p.i. The means and standard errors were plotted. The number of animals were n=5 for LASV LF2384 and n=4 for LASV LF2350 since one guinea pig died after virus inoculation and blood sampling. Organ and blood samples was collected for CBC, virus dissemination, and transcription analyses.
